# Supplementary material for: Parallels in the sequential organization of birdsong and human speech
Source: Nat Commun. 2019 Aug 12;10:3636. doi: 10.1038/s41467-019-11605-y (PMC6690877; doi:10.1038/s41467-019-11605-y)
Supplement: Supplementary file 1 — Supplementary Information [file 41467_2019_11605_MOESM1_ESM.pdf]

Supplementary Information

Parallels in the sequential organization of birdsong and human speech

Tim Sainburg[1,2], Brad Theilman[3], Marvin Thielk[3] & Timothy Q. Gentner[1,3,4,5]

[1]*Department of Psychology, University of California, UC San Diego, La Jolla CA 92093*

[2]*Center for Academic Research & Training in Anthropogeny, UC San Diego, La Jolla CA 92093*

[3]*Neurosciences Graduate Program, University of California, UC San Diego, La Jolla CA 92093*

[4]*Neurobiology Section, Division of Biological Sciences, UC San Diego, La Jolla CA 92093*

[5]*Kavli Institute for Brain and Mind, La Jolla CA 92093*

AICc To calculate the AICc[42] for each competing model, we first calculated the (log scaled) residual sum of squares as:

$$RSS\left(MI, MI_{model}\right) = \left(MI - MI_{model}\right)^2 \tag{9}$$

The log-likelihood of the model can then be calculated as:

$$\log \mathcal{L} = -\frac{n}{2} \log\left(\frac{RSS}{n}\right) \tag{10}$$

where n is the sample size. AIC can then be calculated as:

$$AIC = -2 \log \mathcal{L} + 2K \tag{11}$$

where K is the total number of parameters in the model that can be estimated. To be conservative we used the sample bias corrected AIC, AICc, for all reported results, calculated as:

$$AICc = AIC + \frac{2K(K+1)}{n-K-1} \tag{12}$$

although the correction made no difference in the results. We computed the ΔAIC as the difference between the best-fit model and each other model:

$$\Delta AIC_i = AICc_i - \min(AICc) \tag{13}$$

Using ΔAIC for each model, we calculate the relative likelihood of that model given the data as:

$$\ell_i = \mathcal{L}(model_i \mid data) = e^{-\frac{1}{2}\Delta AIC_i} \tag{14}$$

Then the relative probability of each model given the data is computed as the likelihood of each model over the sum of the likelihood of all competing models:

$$P\left(model_i \mid data\right) = \frac{\ell_i}{\sum_j \ell_j} \tag{15}$$

Finally, the evidence ratio for the best model versus any other given model is the ratio of probabilities of any two given models.

Supplementary Figures

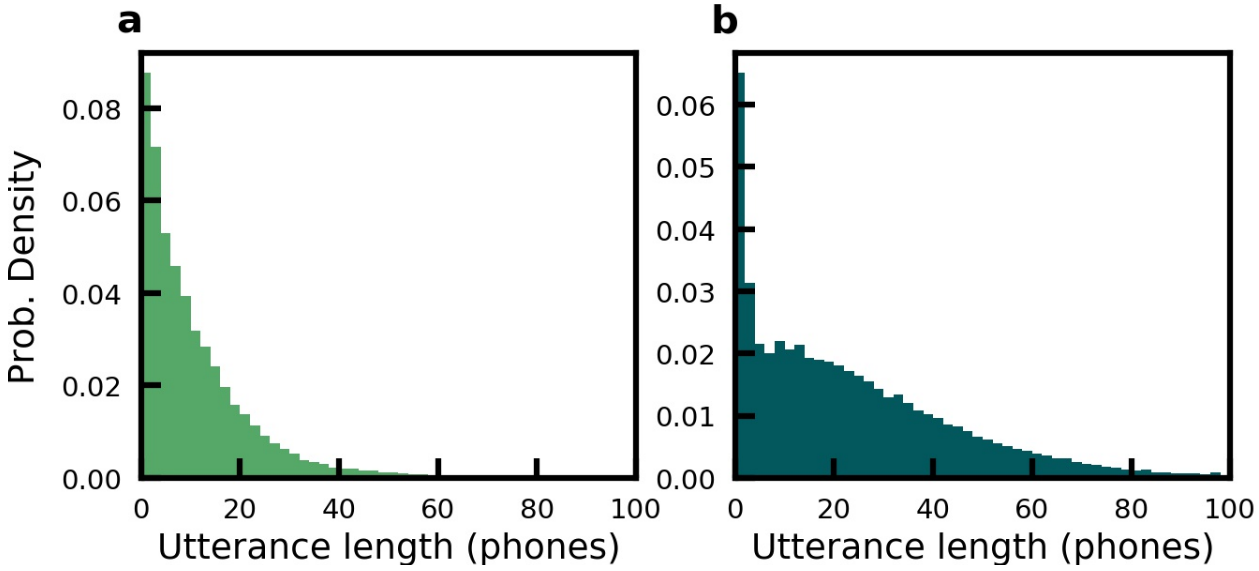

Supplementary Figure 1: Utterance length in phones for English (a) and Japanese (b). The median utterance length in Japanese is 19 phones and in English is 21 phones. The German and Italian data sets were not transcribed by utterance.

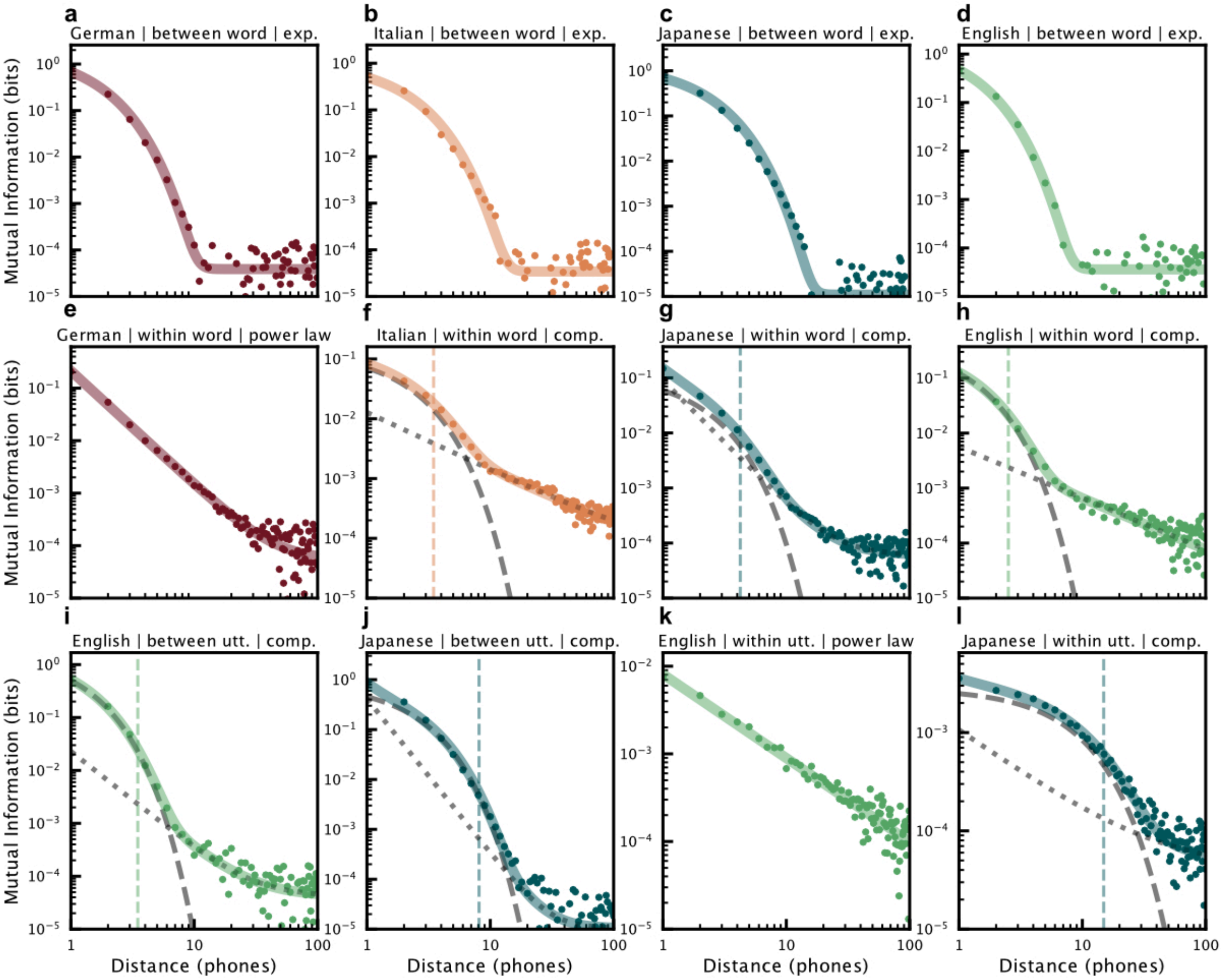

Supplementary Figure 2: MI decay between phones in shuffled speech for different languages (maroon: German, blue-green: Japanese, orange: Italian, green: English). All plots show the MI between phones plotted as a function of sequential distance between phones (as in Figure 3). Panels (a-d) show MI when word order is shuffled while phone order within words is preserved. In all cases, decay is best fit by an exponential model (colored lines). Panels (e-h) show MI when phone order within words is shuffled and word order is preserved. Italian (f), Japanese (g), and English (h) are best fit by a composite model, whereas German (e) is best fit by a power-law model. Panels (i) and (j) show MI when the order of utterances are shuffled and phone order within each utterance is preserved. Both English (i) and Japanese (j) are best fit by a composite decay model. Panels k and l show MI when phone order within utterances is shuffled, and utterance order is preserved. English (k) is best-fit by a power-law model while Japanese (l) is best fit by a composite model.

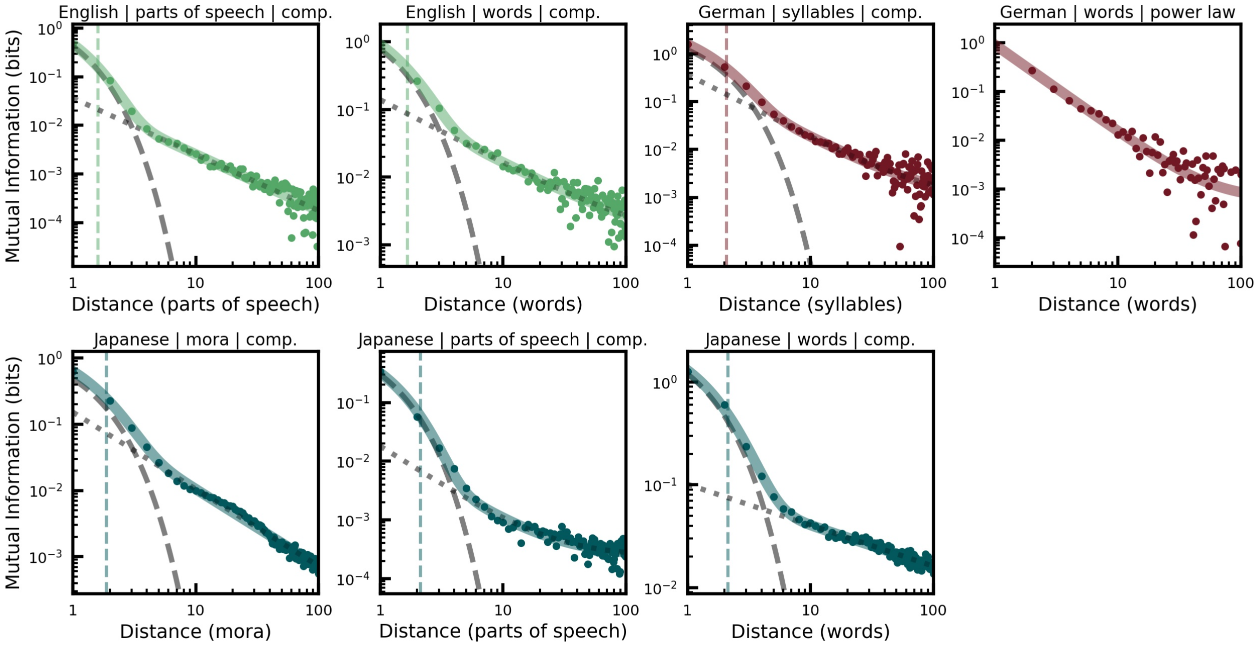

Supplementary Figure 3: MI decay between words, syllables, mora, and parts-of-speech plotted as a function of sequential distances between each of these elements in three languages (green: English, maroon: German, blue-green: Japanese). Not all element categories are available for all languages. For all cases but German words, MI decay is best fit by a composite model (colored lines) with exponential and power-law decays, shown as a dashed and dotted grey lines, respectively. The MI decay between German words is best fit by a power-law. The minima in curvature (colored vertical dashed lines) for words, part-of-speech, and syllables are shorter (in their respective units) than the minima for phones in each language. For English, the minimum curvature is at 1.7 for words, and at 1.6 for parts-of-speech. For German syllables the minimum curvature is at 2.1. For Japanese, the minimum curvature is at 1.9 for mora, 2.1 for words, and 2.1 for parts-of-speech. A minimum curvature is not given for German words because the decay is best fit by a power-law model alone.

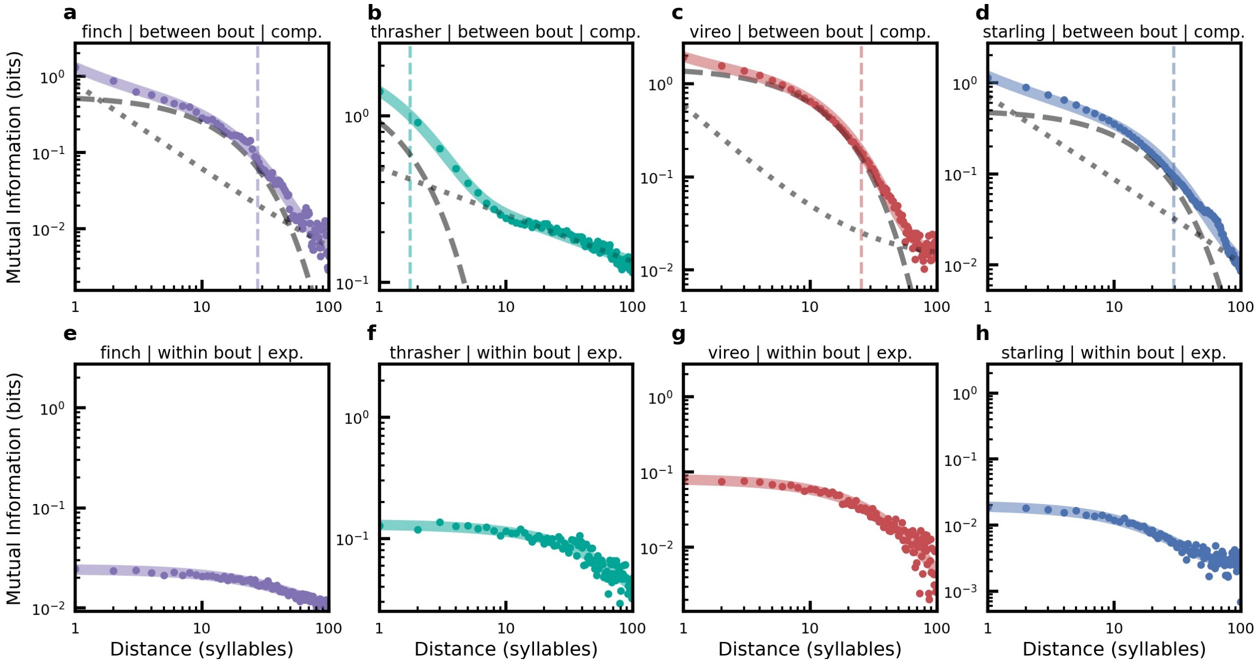

Supplementary Figure 4: MI decay between syllables in shuffled songs from four songbird species (purple: Bengalese finch, teal: California thrasher, red: Cassin's vireo, blue: European starling). All plots show the MI between syllables plotted as a function of sequential distance between syllables (as in Figure 4). Panels (a-d) show MI when bout order is shuffled and syllable order within bouts is preserved. Decay in all species is best fit by a composite model. Panels (e-h) show MI when syllable order within each bout is shuffled, and the order of bouts is preserved. Decay in all species is best fit by an exponential model.

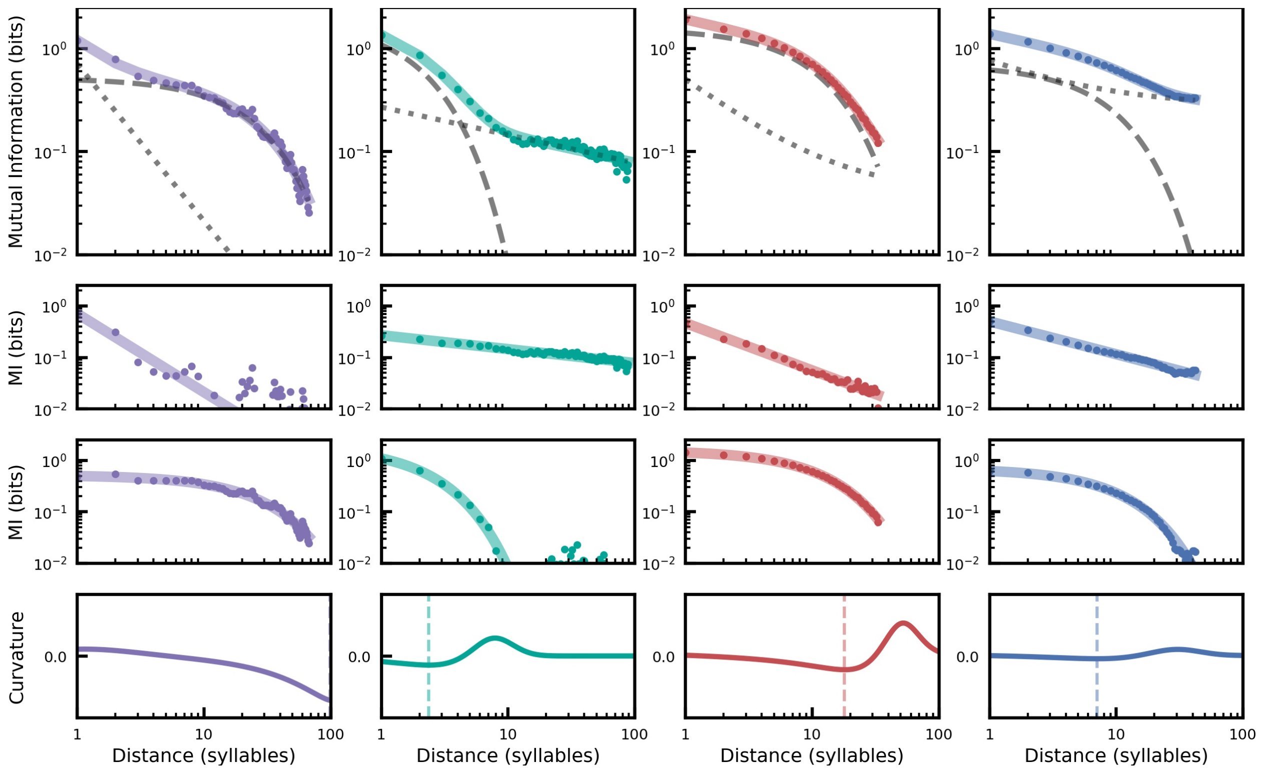

Supplementary Figure 5: Mutual information decay between syllables in the songs of four songbird species (as in Figure 4; purple: Bengalese finch, teal: California thrasher, red: Cassin's vireo, blue: European starling), but when the analysis is restricted to syllable pairs that do not span multiple song bouts. MI is plotted from a distance of 1 syllable to the median song length in syllables, to allow a sufficient number of examples for the MI calculation.

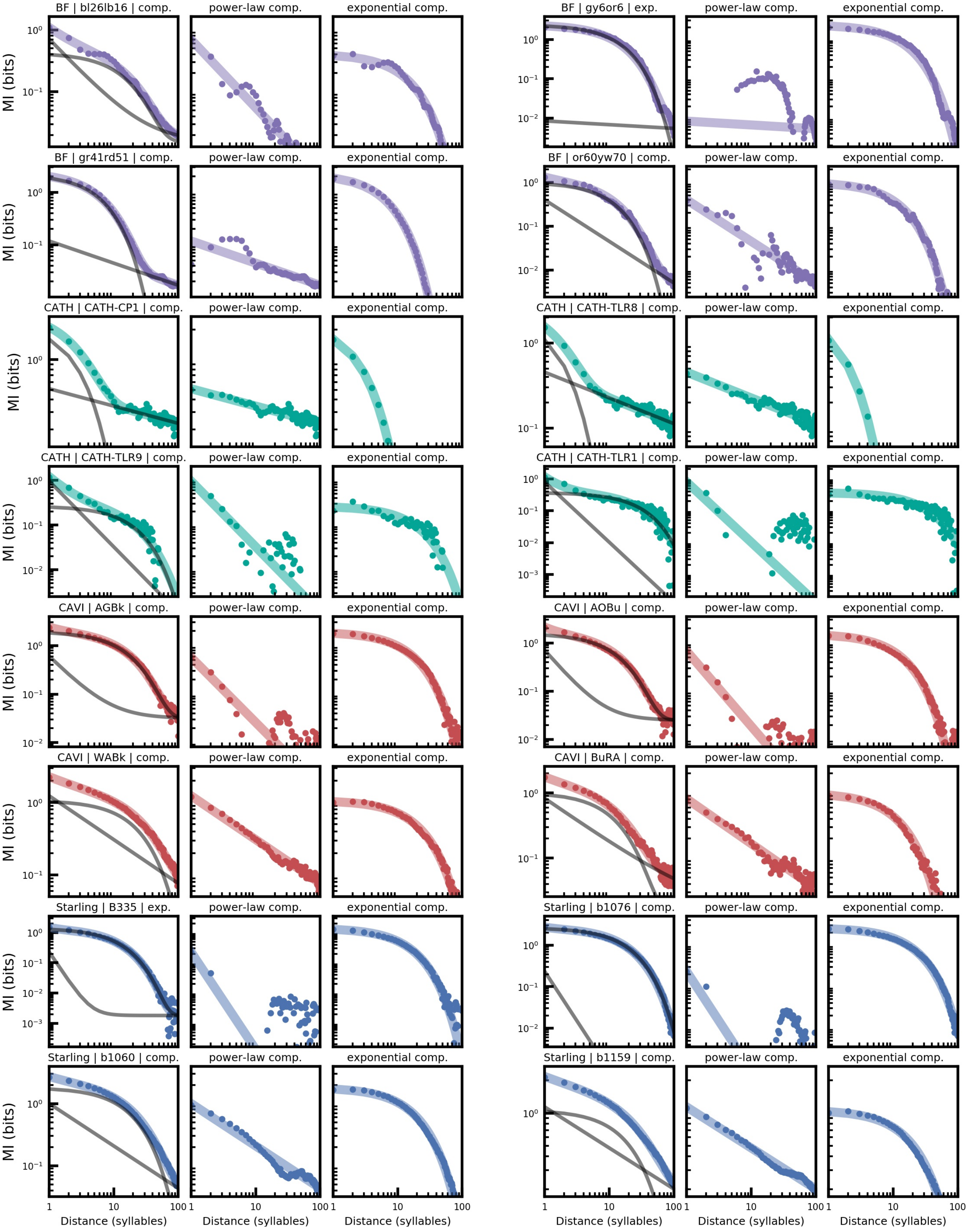
BF | bl26lb16 | comp.
power-law comp.
exponential comp.
BF | gy6or6 | exp.
BF | gr41rd51 | comp.
BF | or60yw70 | comp.
CATH | CATH-CP1 | comp.
CATH | CATH-TLR8 | comp.
CATH | CATH-TLR9 | comp.
CATH | CATH-TLR1 | comp.
CAVI | AGBk | comp.
CAVI | AOBu | comp.
CAVI | WABk | comp.
CAVI | BuRA | comp.
Starling | B335 | exp.
Starling | b1076 | comp.
Starling | b1060 | comp.
Starling | b1159 | comp.
MI (bits)
Distance (syllables)

Supplementary Figure 6: MI decay in the four largest data sets from individual songbirds in each species. Plots are grouped into sets of three (in a row), corresponding to the data from a one individual songbird (purple: Bengalese finch, teal: California thrasher, red: Cassin's vireo, blue: European starling). For a given bird, the three subplots from left to right show (1) the full MI decay with the fitted model (colored line) and the individual model components (grey lines), (2) the power-law fit to the MI when the exponential component is subtracted, and (3) exponential fit to the MI when the power-law component is subtracted. The species, individual ID, and best-fit model is shown in the title of the leftmost subplot.

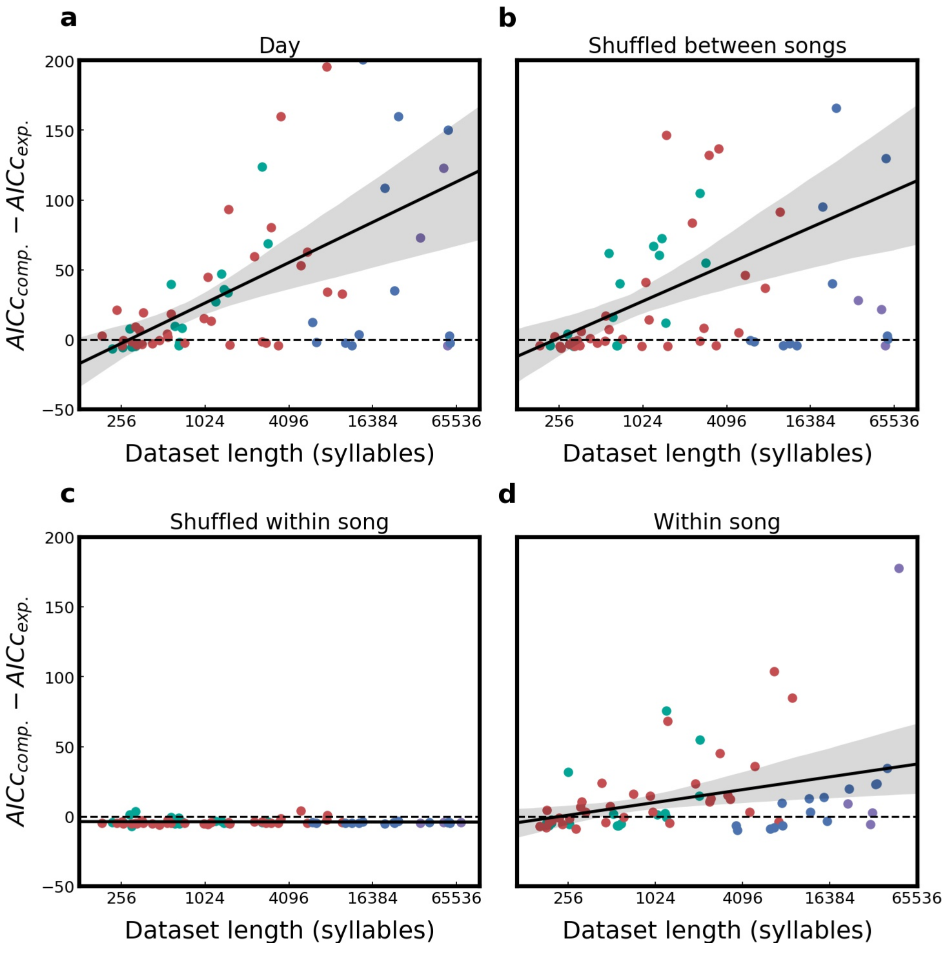

Supplementary Figure 7: Relative decay model fits. Scatterplots (a-d) showing the difference in model fit (ΔAICc) for the composite model versus the exponential model of MI decay for individual songbirds (purple: Bengalese finch, teal: California thrasher, red: Cassin's vireo, blue: European starling) plotted as a function of the number of syllables in each individual songbird's data set. The black line shows a linear regression model with 95% confidence interval fit to the positive relationship between the improvement of the composite model over the exponential model as a function of (log) data set size. Points above zero (dashed line) are better fit by the composite model, while points below are better fit by the exponential model. (a) MI decay for each bird computed across all bouts within a day. (b) The same plot as in (a), but shuffling the ordering of bouts to remove between-bout structure. (c) The same plot as in (a), but shuffling syllable order within bouts, to remove within-bout structure. (d) The same plot as in (a), but where the analysis is restricted to only those syllable pairs within the same song bout.

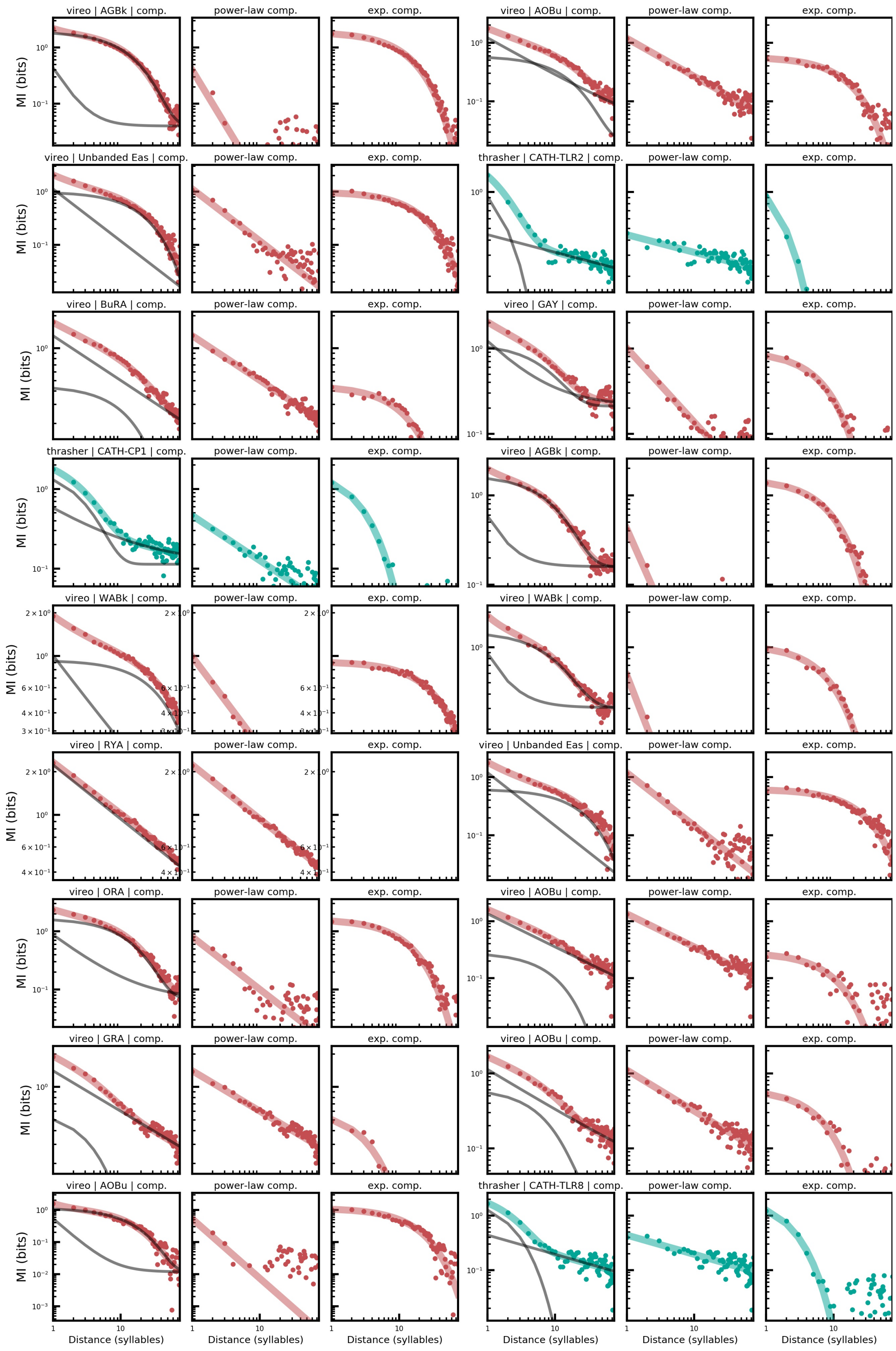
vireo | AGBk | comp.
power-law comp.
exp. comp.
vireo | AOBu | comp.
power-law comp.
exp. comp.
vireo | Unbanded Eas | comp.
power-law comp.
exp. comp.
thrasher | CATH-TLR2 | comp.
power-law comp.
exp. comp.
vireo | BuRA | comp.
power-law comp.
exp. comp.
vireo | GAY | comp.
power-law comp.
exp. comp.
thrasher | CATH-CP1 | comp.
power-law comp.
exp. comp.
vireo | AGBk | comp.
power-law comp.
exp. comp.
vireo | WABk | comp.
power-law comp.
exp. comp.
vireo | WABk | comp.
power-law comp.
exp. comp.
vireo | RYA | comp.
power-law comp.
exp. comp.
vireo | Unbanded Eas | comp.
power-law comp.
exp. comp.
vireo | ORA | comp.
power-law comp.
exp. comp.
vireo | AOBu | comp.
power-law comp.
exp. comp.
vireo | GRA | comp.
power-law comp.
exp. comp.
vireo | AOBu | comp.
power-law comp.
exp. comp.
vireo | AOBu | comp.
power-law comp.
exp. comp.
thrasher | CATH-TLR8 | comp.
power-law comp.
exp. comp.
MI (bits)
Distance (syllables)

Supplementary Figure 8: The decay in MI between syllables in the 18 individual songbirds with the longest available recordings in all data sets. Each set of three plots is from either a Cassin's vireo (red) or California thrasher (teal). The three subplots for each bird are organized as in Supplementary Figure 6, showing (from left to right) the full MI decay (colored line, with individual model components in grey), power-law fit after the exponential component is subtracted, and exponential fit after the power-law component subtracted. The species, individual ID, and best-fit model is given in the title of the left-most subplot.

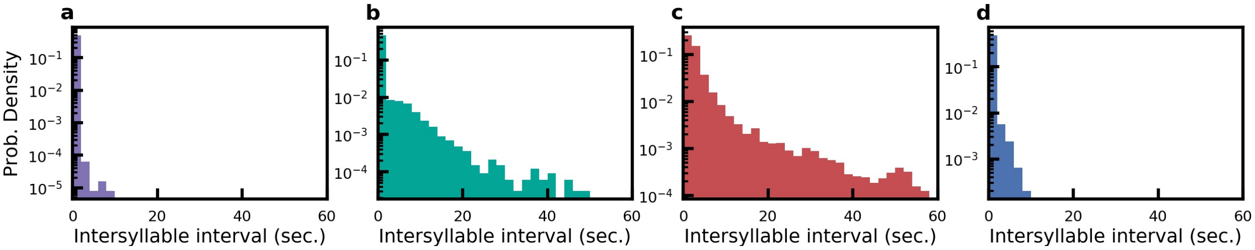

Supplementary Figure 9: The intersyllable interval time in seconds for each songbird species. (a) Bengalese finch (b) California thrasher (c) Cassin's vireo (d) European starling.

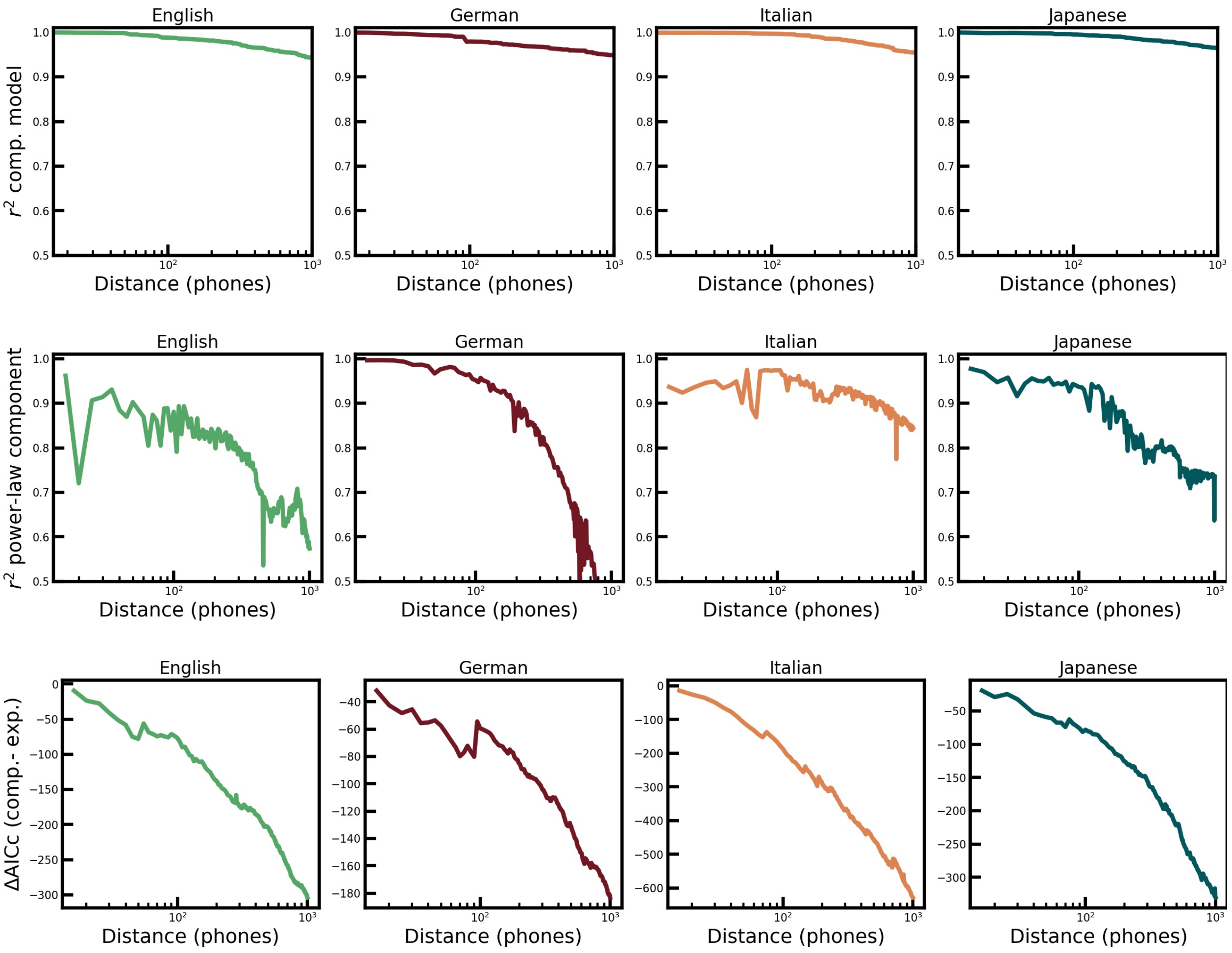

Supplementary Figure 10: The goodness of fit of the composite decay model for each language as a function of the MI analysis length. The coefficient of determination (r^2) for the full composite model (top) and the power-law component of the composite model (center). r^2 is computed for fits of the composite model on MI decay at distances of 15-1000 phones (x-axis). (bottom) ΔAICc between composite and exponential decay models for each language as a function of the maximum phone-to-phone distance computed (green: English, maroon: German, orange: Italian, blue-green: Japanese).

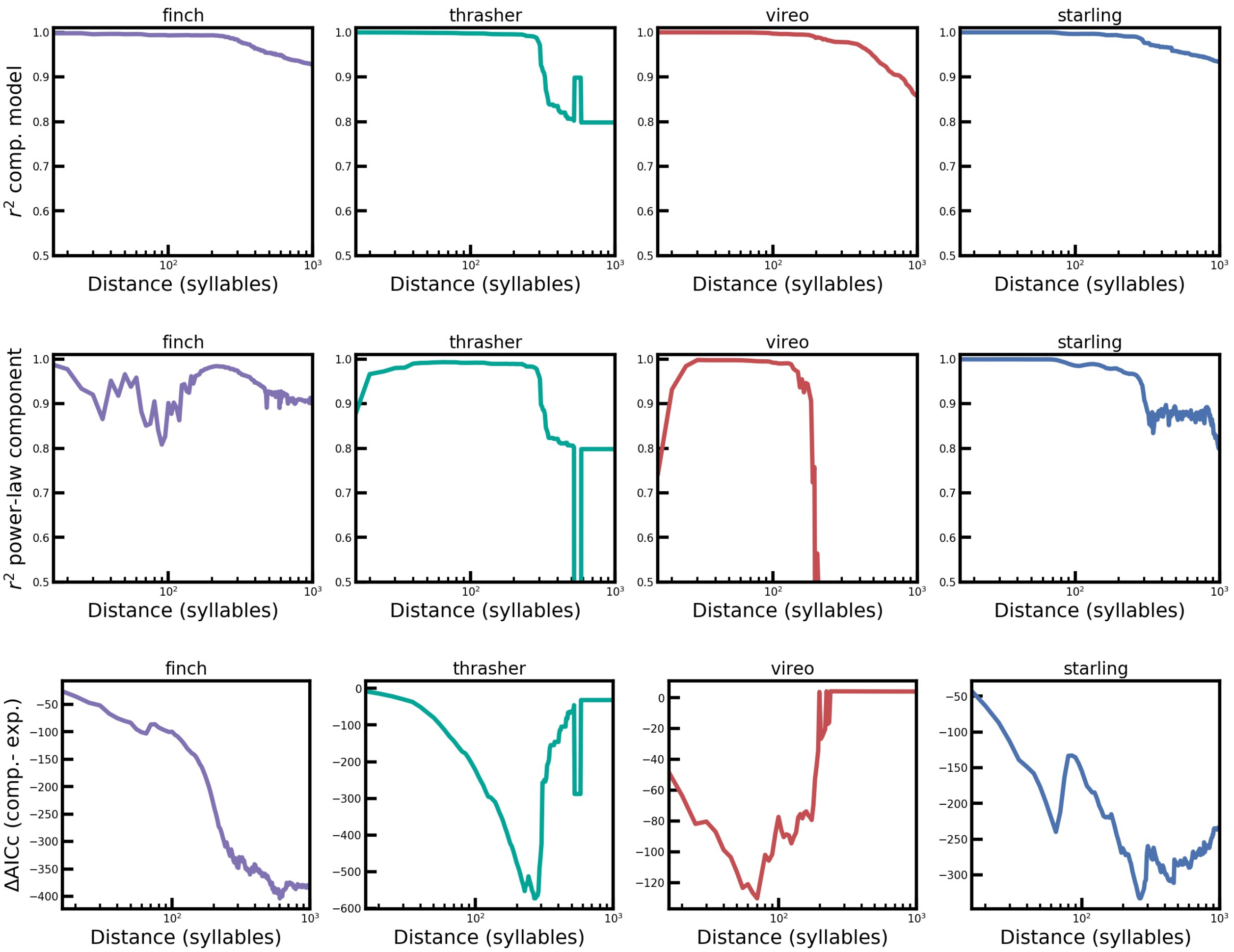

Supplementary Figure 11: The goodness of fit of the composite decay model for each songbird species as a function of the MI analysis length. The coefficient of determination (r^2) for the full composite decay model (top) and the power-law component of the composite model (center). r^2 is computed for fits of the composite model on MI decay of distances of 15-1000 syllables (x-axis). (bottom) ΔAICc between composite and exponential decay models for each species as a function of maximum syllable-to-syllable distance computed (purple: Bengalese finch, teal: California thrasher, red: Cassin's vireo, blue: European starling).

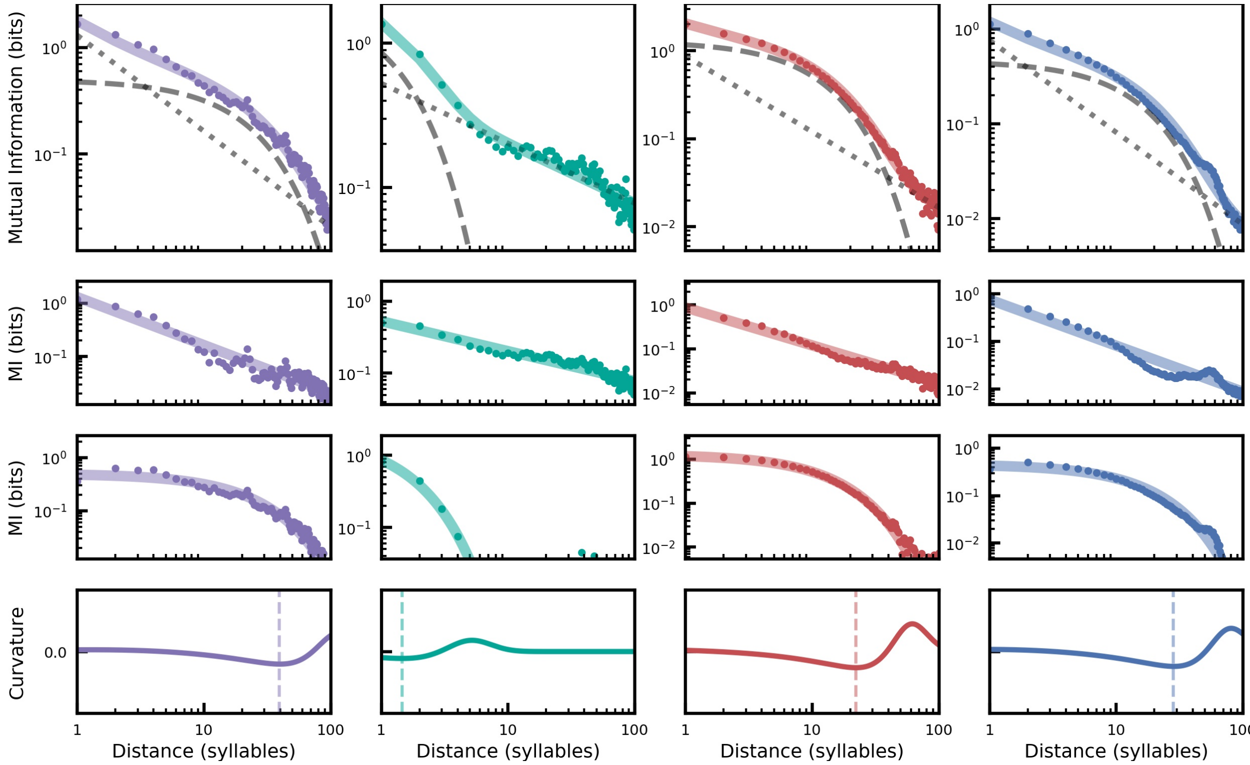

Supplementary Figure 12: Decay of MI between syllables in the birdsong data sets after removing sequentially repeated syllables. Data follow those in Figure 4 (purple: Bengalese finch, teal: California thrasher, red: Cassin's vireo, blue: European starling). The decay of Cassin's vireo, California thrasher, and European starling song is largely unaffected, whereas exponential portion of the decay of Bengalese finch song is shifted.

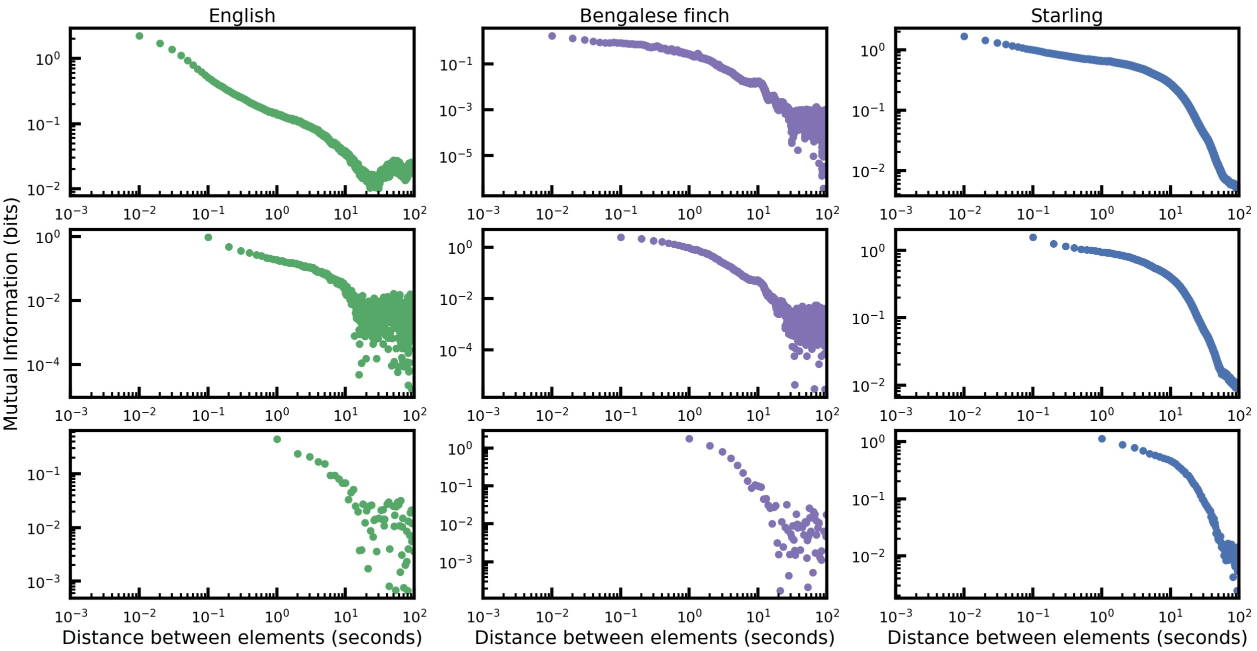

Supplementary Figure 13: Decay in MI between song and speech signal components arbitrarily parsed at multiple timescales. Raw waveforms are split into discrete units at three different timescales (0.01, 0.1, and 1 second), and classified using k-means clustering. Each set of three plots in a column shows the MI between units at one of three timescales (0.001 to 1 second, top to bottom) as a function of the distance between units. Each column shows data for vocalizations of a single individual (green: English, purple: Bengalese finch, blue: European starling). The analysis was only performed on a subset of individuals/data due to the length of time required to segment, cluster and calculate MI on small timescales with large data sets.

Supplementary Tables

**Species**	**Ben. finch**	**Eur. starling**	**Cass. vireo**	**Cal. thrasher**
**Origin**	Laboratory	Wild-caught	Wild	Wild
**# individuals**	4	14	50	18
**# syllables**	215,740	368,956	68,157	15,764
**Duration (hrs.)**	7.44	89.14	94.07	4.5
**Hand Labelled**	Yes	No	Yes	Yes
**Unique syllables (median)**	18.5	151.5	48	51.5
**Syllables in bout (median)**	68	42	7	21
**Syllable length (s; median)**	0.07	0.68	0.33	0.15

Supplementary Table 1: Birdsong dataset statistics.

Dataset	Buckeye	GECO	AsiCA	CSJ
**Language**	English	German	Italian	Japanese
**Transcripts**	40	92	61	201
**Duration (Hrs.)**	37.9	39.9	35.4	37.6
**# Phones**	841,266	839,543	1,065,084	1,633,659
**Unique phone labels**	45	70	90	49
**Transcription**				
**Phone**	Yes	Yes	Ortho-phonetic	Yes
**Mora**	No	No	No	Yes
**Words**	Yes	Yes	Ortho-phonetic	Yes
**Syllables**	No	Yes	No	No
**Part of speech**	Yes	No	No	Yes
**Utterance**	Yes	No	No	Yes

Supplementary Table 2: Language dataset statistics.

		German	Italian	English	Japanese
**AICc**	**exp**	-261.645	-355.721	-255.784	-401.333
	**composite**	-343.68	-566.454	-311.642	-509.903
	**power-law**	-326.137	-435.96	-279.64	-348.479
r^2	**exp**	0.966	0.977	0.954	0.991
	**composite**	0.986	0.997	0.975	0.997
	**power-law**	0.983	0.99	0.964	0.985
**Relative likelihood**	**exp**	<0.001	<0.001	<0.001	<0.001
	**composite**	>0.999	>0.999	>0.999	>0.999
	**power-law**	<0.001	<0.001	<0.001	<0.001
**Relative probability**	**exp**	<0.001	<0.001	<0.001	<0.001
	**composite**	>0.999	>0.999	>0.999	>0.999
	**power-law**	<0.001	<0.001	<0.001	<0.001

Supplementary Table 3: Language corpus model fit results at 100 phones of distance.

		Ben. finch	Cal. thrasher	Cass. vireo	Eur. starling
**AICc**	**exp**	-489.251	-582.14	-637.678	-520.903
	**composite**	-586.509	-797.431	-763.787	-676.984
	**power-law**	-390.009	-698.559	-354.734	-405.85
r^2	**exp**	0.98	0.975	0.995	0.981
	**composite**	0.993	0.997	0.999	0.996
	**power-law**	0.945	0.992	0.92	0.942
**Relative likelihood**	**exp**	<0.001	<0.001	<0.001	<0.001
	**composite**	>0.999	>0.999	>0.999	>0.999
	**power-law**	<0.001	<0.001	<0.001	<0.001
**Relative probability**	**exp**	<0.001	<0.001	<0.001	<0.001
	**composite**	>0.999	>0.999	>0.999	>0.999
	**power-law**	<0.001	<0.001	<0.001	<0.001

Supplementary Table 4: Birdsong dataset model fit results at 100 syllables of distance.
