# Supplementary material for: Parallels in the sequential organization of birdsong and human speech
Source: Nat Commun. 2019 Aug 12;10:3636. doi: 10.1038/s41467-019-11605-y (PMC6690877; doi:10.1038/s41467-019-11605-y)
Supplement: Supplementary file 3 — Reporting Summary [file 41467_2019_11605_MOESM3_ESM.pdf]

nature research

Corresponding author(s): Timothy Q Gentner, Tim Sainburg

Reporting Summary

Nature Research wishes to improve the reproducibility of the work that we publish. This form provides structure for consistency and transparency in reporting. For further information on Nature Research policies, see Authors & Referees and the Editorial Policy Checklist.

Statistical parameters

When statistical analyses are reported, confirm that the following items are present in the relevant location (e.g. figure legend, table legend, main text, or Methods section).

n/a	Confirmed	
☐	☒	The exact sample size (*n*) for each experimental group/condition, given as a discrete number and unit of measurement
☐	☒	An indication of whether measurements were taken from distinct samples or whether the same sample was measured repeatedly
☐	☒	The statistical test(s) used AND whether they are one- or two-sided<br>*Only common tests should be described solely by name; describe more complex techniques in the Methods section.*
☐	☒	A description of all covariates tested
☐	☒	A description of any assumptions or corrections, such as tests of normality and adjustment for multiple comparisons
☐	☒	A full description of the statistics including central tendency (e.g. means) or other basic estimates (e.g. regression coefficient) AND variation (e.g. standard deviation) or associated estimates of uncertainty (e.g. confidence intervals)
☐	☒	For null hypothesis testing, the test statistic (e.g. *F*, *t*, *r*) with confidence intervals, effect sizes, degrees of freedom and *P* value noted<br>*Give P values as exact values whenever suitable.*
☐	☒	For Bayesian analysis, information on the choice of priors and Markov chain Monte Carlo settings
☐	☒	For hierarchical and complex designs, identification of the appropriate level for tests and full reporting of outcomes
☒	☐	Estimates of effect sizes (e.g. Cohen's *d*, Pearson's *r*), indicating how they were calculated
☐	☒	Clearly defined error bars<br>*State explicitly what error bars represent (e.g. SD, SE, CI)*

Our web collection on statistics for biologists may be useful.

Software and code

Policy information about availability of computer code

Data collection	Starling songs were collected using arecord (version 1.0.27.2) running in Ubuntu 14.0.4. Bengalese finch and Cassin's vireo songs were gifted from other labs, as noted in the text.
Data analysis	Analyses were done with custom software written in Python (versoin 2 and 3) and Tensorflow, in Jupyter notebooks. All code is available on GitHub at https://github.com/timsainb/ParallelsBirdsongLanguagePaper

For manuscripts utilizing custom algorithms or software that are central to the research but not yet described in published literature, software must be made available to editors/reviewers upon request. We strongly encourage code deposition in a community repository (e.g. GitHub). See the Nature Research guidelines for submitting code & software for further information.

Data

Policy information about availability of data

All manuscripts must include a data availability statement. This statement should provide the following information, where applicable:

- Accession codes, unique identifiers, or web links for publicly available datasets
- A list of figures that have associated raw data
- A description of any restrictions on data availability

The software created for all of the analyses performed in this article will be made available open source and online at {https://github.com/timsainb/GenerativeVocalizationNetwork}{GitHub.com/timsainb/GenerativeVocalizationNetwork} upon publication. The European starling song dataset is available from the authors upon reasonable request. The availability of the Cassin's vireo and Bengalese finch datasets is at the discretion of the corresponding laboratories. All analysis tools are available open source and available at the GitHub permalink. Specific scripts or generated sequence corpus' corresponding to the individual birds used in this analysis are also available upon reasonable request.

The citations for each publicly available dataset are given in the methods section.
The buckeye corpus was downloaded from (https://buckeyecorpus.osu.edu/).
Oyanoya corpus was downloaded from: http://journals.plos.org/plosone/article?id=10.1371/journal.pone.0159188
Cassin's song dataset was downloaded from: https://www.sciencedirect.com/science/article/pii/S1574954115000151

Field-specific reporting

Please select the best fit for your research. If you are not sure, read the appropriate sections before making your selection.

☒ Life sciences ☐ Behavioural & social sciences ☐ Ecological, evolutionary & environmental sciences

For a reference copy of the document with all sections, see nature.com/authors/policies/ReportingSummary-flat.pdf

Life sciences study design

All studies must disclose on these points even when the disclosure is negative.

Sample size	Vocalizations from three species of songbirds, and human speech were used for the analyses. For each songbird species' dataset, the maximum number of individuals and vocalizations available were used. Only the European starling dataset was collected in our lab. The human speech dataset is a freely available speech corpus that includes speech from 40 participants. Of the 40 participants in the full corpus, all were used in our study, and the entire dataset of phonemes for each speaker was analyzed and included in the reported results. For the three songbird species, we chose datasets that were the largest available in terms of the number of syllables in the dataset. For Starlings and Cassin's Vireo, we used datasets with over 10,000 syllables for each singer. For Begalese finches, we used datasets with 4,000 or more syllables, due to the comparatively smaller syllable repertoires of individual Bengalese finches.
Data exclusions	One European starling was discluded from the curvature analysis, which was identified through a commonly used outlier analysis described in the Methods section, and reference 48.
Replication	As the methods used in this study are purely computational, the analysis is fully replicable upon the waveform datasets using the code repository provided.
Randomization	Randomization is not performed, as different groups correspond to different species.
Blinding	Blinding is not relevant to this study, as different groups correspond to different species and all analyses are objective.

Reporting for specific materials, systems and methods

Materials & experimental systems

n/a	Involved in the study
☒	☐ Unique biological materials
☒	☐ Antibodies
☒	☐ Eukaryotic cell lines
☒	☐ Palaeontology
☐	☒ Animals and other organisms
☒	☐ Human research participants

Methods

n/a	Involved in the study
☒	☐ ChIP-seq
☒	☐ Flow cytometry
☒	☐ MRI-based neuroimaging

Animals and other organisms

Policy information about studies involving animals; ARRIVE guidelines recommended for reporting animal research

Laboratory animals	Both Bengalese finch datasets were recorded in laboratory animals and are obtained from males (>120 days post-hatch).
Wild animals	The European starling datasets were acquired from wild caught male animals recorded in-captivity in sound isolated chambers. Sexing was done by morphological characteristics. Animals were wild caught in Southern California, and transported to and housed in outdoor aviaries prior to recording. All subjects were >1 year of age at the time of capture, as indicated by plumage.
Field-collected samples	The Cassin's vireo dataset consists entirely of males, and was collected in the wild from the Sierra Nevada mountains in California.
